# Dose-Dependent AMPK-Dependent and Independent Mechanisms of Berberine and Metformin Inhibition of mTORC1, ERK, DNA Synthesis and Proliferation in Pancreatic Cancer Cells

**DOI:** 10.1371/journal.pone.0114573

**Published:** 2014-12-10

**Authors:** Ming Ming, James Sinnett-Smith, Jia Wang, Heloisa P. Soares, Steven H. Young, Guido Eibl, Enrique Rozengurt

**Affiliations:** 1 Division of Digestive Diseases, Department of Medicine, David Geffen School of Medicine, University of California Los Angeles, Los Angeles, California, United States of America; 2 Division of Hematology-Oncology, Department of Medicine David Geffen School of Medicine, University of California Los Angeles, Los Angeles, California, United States of America; 3 Department of Surgery, David Geffen School of Medicine, University of California Los Angeles, Los Angeles, California, United States of America; 4 CURE: Digestive Diseases Research Center, David Geffen School of Medicine, University of California Los Angeles, Los Angeles, California, United States of America; 5 Molecular Biology Institute, University of California Los Angeles, Los Angeles, California, United States of America; Peter MacCallum Cancer Centre, Australia

## Abstract

Natural products represent a rich reservoir of potential small chemical molecules exhibiting anti-proliferative and chemopreventive properties. Here, we show that treatment of pancreatic ductal adenocarcinoma (PDAC) cells (PANC-1, MiaPaCa-2) with the isoquinoline alkaloid berberine (0.3–6 µM) inhibited DNA synthesis and proliferation of these cells and delay the progression of their cell cycle in G1. Berberine treatment also reduced (by 70%) the growth of MiaPaCa-2 cell growth when implanted into the flanks of nu/nu mice. Mechanistic studies revealed that berberine decreased mitochondrial membrane potential and intracellular ATP levels and induced potent AMPK activation, as shown by phosphorylation of AMPK α subunit at Thr-172 and acetyl-CoA carboxylase (ACC) at Ser^79^. Furthermore, berberine dose-dependently inhibited mTORC1 (phosphorylation of S6K at Thr^389^ and S6 at Ser^240/244^) and ERK activation in PDAC cells stimulated by insulin and neurotensin or fetal bovine serum. Knockdown of α_1_ and α_2_ catalytic subunit expression of AMPK reversed the inhibitory effect produced by treatment with low concentrations of berberine on mTORC1, ERK and DNA synthesis in PDAC cells. However, at higher concentrations, berberine inhibited mitogenic signaling (mTORC1 and ERK) and DNA synthesis through an AMPK-independent mechanism. Similar results were obtained with metformin used at doses that induced either modest or pronounced reductions in intracellular ATP levels, which were virtually identical to the decreases in ATP levels obtained in response to berberine. We propose that berberine and metformin inhibit mitogenic signaling in PDAC cells through dose-dependent AMPK-dependent and independent pathways.

## Introduction

Pancreatic ductal adenocarcinoma (PDAC) is a devastating disease, with overall 5-year survival rate of only 6% [1]. The incidence of this disease in the US is estimated to increase to more than 44,000 new cases in 2014 and is now the fourth leading cause of cancer mortality in both men and women [2]. Total deaths due to PDAC are projected to increase dramatically to become the second leading cause of cancer-related deaths before 2030 [1] As the current therapies offer very limited survival benefits, novel strategies to treat and prevent this aggressive disease are urgently required [3].

G protein-coupled receptors (GPCRs) and their cognate agonists are increasingly implicated as autocrine/paracrine growth factors for multiple solid tumors, including small cell lung cancer, colon, prostate, breast and pancreas [4]–[8]. We showed that pancreatic cancer cell lines express multiple GPCRs [9] and a variety of GPCR agonists, including neurotensin, angiotensin II and bradykinin, stimulated DNA synthesis in pancreatic cancer cell lines, including PANC-1 and MiaPaca-2 [9]–[12]. Furthermore, a broad-spectrum GPCR antagonist [13], [14], inhibited the growth of pancreatic cancer cells either *in vitro* or xenografted into nu/nu mice [15]. Other studies demonstrated increased expression of GPCRs in pancreatic cancer tissues [16]–[19]. Subsequently, we identified positive crosstalk between insulin/IGFI receptors and GPCR signaling systems in pancreatic cancer cells, leading to mTORC1 signaling and ERK activation, and synergistic stimulation of DNA synthesis and cell proliferation [20]–[22]. These findings assume an added importance in view of the large number of epidemiological studies linking long standing type-2 diabetes mellitus (T2DM), obesity and metabolic syndrome, characterized by peripheral insulin resistance and compensatory overproduction of insulin, with increased risk for developing pancreatic cancer [23]–[32].

The biguanide metformin (1,1-dimethylbiguanide hydrochloride) derived from galegine, a phytochemical from *Galega officinalis,* is the most widely prescribed drug for treatment of T2DM, *worldwide* [33], [34]. Systemically, metformin lowers blood glucose levels through reduced hepatic gluconeogenesis, increases glucose uptake in skeletal muscles and adipose tissue [34], [35] and reduces the circulating levels of insulin and IGF-1 [36], [37]. At the cellular level, metformin indirectly stimulates AMP–activated protein kinase (AMPK) activation [38] via inhibition of mitochondrial function, though other mechanisms of metformin action have been also suggested at high doses [39]. Major downstream targets of AMPK include TSC2 and Raptor [40]–[43]. The AMPK-mediated phosphorylation of these targets inhibits mTOR complex 1 (mTORC1) activity in a variety of cell types, including PDAC cells [44], [45] and disrupts positive crosstalk between insulin/IGFI receptors and GPCR signaling systems [21], [46]. Interestingly, a number of observational studies suggest that metformin reduces incidence and improved prognosis of a variety of cancers in patients with T2DM [47], [48], though this this conclusion is under scrutiny [49]. In the setting of PDAC, diabetic patients who had received metformin appear to have lower adjusted incidence and better survival compared with those who had not taken metformin or used other anti-diabetic agents [47], [50]–[52]. We hypothesized that structurally unrelated natural or synthetic compounds that interfere with mitochondrial-mediated ATP synthesis and target mTORC1 and ERK pathways, could provide novel anti-PDAC agents.

Natural products represent a rich reservoir of potential small chemical molecules exhibiting diverse pharmacological properties. The isoquinoline alkaloid berberine [53]–[55], a phytochemical extracted from a variety of medicinal plants, including plants of the *Berberis* species induces multiple biological effects, including anti-obesity, anti-diabetic, anti-cancer and calorie-restriction effects [55]–[62]. The cellular mechanism(s) involved, however, remains incompletely understood. Berberine has been reported to inhibit mitochondrial function and induce AMPK activation [63] but other mechanisms of action of this alkaloid have been proposed when added at high concentrations [64], [65]. Despite its potential clinical implications, there is no understanding of the precise mechanism(s) by which berberine inhibits the proliferation of cancer cells and it is not known whether this agent has any direct effect on signaling and proliferation of PDAC cells harboring *KRAS* mutations, characteristic of >90% of ductal pancreatic carcinomas.

In this study, we show that berberine inhibits DNA synthesis, cell cycle progression and proliferation in PANC-1 and MiaPaca-2 pancreatic cancer cells. Furthermore, berberine administration inhibits the growth of PDAC tumor xenografts *in vivo* as effectively as metformin. In mechanistic studies, we demonstrate that berberine, like metformin decreases mitochondrial membrane potential and ATP levels and concomitantly induces AMPK activation. Based on results using siRNA-mediated knockdown of AMPK, we propose that the inhibitory effects of berberine and metformin are mediated through AMPK-dependent and AMPK-independent pathways depending on the dose of each agent. This conclusion provides a plausible explanation for apparently contradictory reports on the role of AMPK in the mechanism of action of berberine and metformin in other model systems.

## Materials and Methods

### Chemicals and Reagents

Dulbecco’s modified Eagle Medium (DMEM) was obtained from Invitrogen (Carlsbad, CA). Neurotensin, insulin, berberine and metformin were obtained from Sigma Chemical (St. Louis, MO). All antibodies were purchased from Cell Signaling Technology (Danvers, MA). Horseradish peroxidase-conjugated anti-rabbit IgG and anti-mouse IgG were from GE Healthcare Bio-Sciences Corp (Piscataway, NJ). All other reagents were of the highest grade available.

### Cells and Culture Conditions

The human pancreatic cancer cell lines PANC-1 and MiaPaCa-2 were obtained from the American Type Culture Collection (ATCC, Manassas, VA). These cell lines were chosen because they harbor mutations typical of human pancreatic cancer [66], including activating mutations in KRAS, TP53 (encoding the p53 protein) and CDKN2A (also known as p16 or p16INK4a). Indeed, there is an excellent correlation between point mutation frequencies in PDAC cell lines and primary tumors [67]. Cells were grown in DMEM supplemented with 2 mM glutamine, 1 mM Na-pyruvate, 100 units/mL penicillin, and 100 µg/mL streptomycin and 10% fetal bovine serum (FBS) at 37°C in a humidified atmosphere containing 10% CO2. In the experiments, the glucose concentration in DMEM was adjusted to 5 mM, a physiological level in human serum.

### [^3^H]-Thymidine Incorporation into DNA

PANC-1 and MiaPaCa-2 cells (1×10^5^) were plated and grown in 3.5 cm tissue culture plates for 5 days in DMEM supplemented with 10% FBS. The cells were washed twice and incubated for 24 h with DMEM containing 5 mM glucose and 1% FBS. To start the experiment, fresh medium containing the specified concentration of agonist and/or inhibitor was added after washing twice with DMEM (4 cultures used for each condition), and then the cells were incubated for 17 h and then pulse labeled for 6 h with [^3^H]-thymidine (0.25 µCi/ml). The cells were fixed with 5% trichloroacetic acid and washed twice with ethanol. Acid-insoluble pools were dissolved in 0.1 N NaOH with 1% SDS and the radioactivity incorporated was counted in a liquid scintillation counter.

### Mitochondrial Membrane Potential

The cell-permeable JC-1 dye (Invitrogen), which exhibits potential-dependent accumulation in the mitochondria, was used as an indicator of mitochondrial membrane potential. Following treatment without or with berberine or metformin, JC-1 was added to cultures of PANC-1 or MIA PaCa-2 cells at 1 µg/ml for 30 min. Then, the media was exchanged for Hanks Buffered Saline Solution containing 5 mM glucose and cells were immediately imaged using rhodamine and fluorescein optics. Images were stored from several visual fields. The histogram analysis feature of Photoshop (Adobe) was used to measure the average red and average green fluorescence intensity from about 50 cells in a visual field. At least 5 independent fields were measured in each condition. The results are expressed as an average ratio of red/green florescent intensity in a single visual field (mean ± SEM). The ratio of red/green fluorescence intensity indicates mitochondrial membrane potential with a decreased ratio indicating a loss of potential.

### ATP Determination

PANC-1 and MiaPaCa-2 cells (5×10^4^) were plated and grown in 24 well culture plates for 5 days in DMEM and 10% FBS. The cells were then incubated for 24 h with DMEM containing 5 mM glucose and 1% FBS. The cells were washed twice with DMEM containing 5 mM glucose and incubated in serum-free medium for 17 h in the absence or presence of berberine or metformin. ATP levels were determined using the firefly luciferase/D-luciferin ATP determination Kit according to the manufacturer’s protocol (Life Technologies Grand Island, NY).

### Knockdown of AMPK levels via siRNA transfection

Silencer Select siRNAs were purchased from Life Technologies (Grand Island, NY) and designed to target either human AMPK_α1_ or human AMPK_α2_. Cells were transfected using the reverse transfection method. Either Silencer Select non-targeting negative control or a mixture of 10 nM AMPK_α1_ and 10 nM AMPK_α2_ siRNA (AMPKα1, α2 siRNA) were mixed with Lipofectamine RNAi MAX (Life Technologies Grand Island, NY) according to the manufacturer’s protocol and added to 24 well plates. PANC-1 cells were then plated on top of the siRNA/Lipofectamine RNAiMAX complex at a density of 10^5^ cells/well.in DMEM containing 5 mM glucose and 10% FBS. Three days after transfection the cells were incubated for 24 h with DMEM containing 5 mM glucose and 1% FBS. The cells were washed twice with DMEM containing 5 mM glucose and incubated in serum-free medium for 17 h in the absence or presence of berberine or metformin and then treated as described in the corresponding figure legends.

### Flow cytometric/cell cycle analysis

The proportion of cells in the G_0_/G_1_, S, G_2_, and M phases of the cell cycle was determined by flow cytometric analysis. PANC-1 cells (2×10^4^ cells) were seeded in DMEM containing 2.5% FBS. After 24 h the cultures were treated without or with berberine in medium containing 2.5% FBS for 3 days. Cells were then harvested by trypsinization, centrifuged at 1,000 *g* for 5 min and resuspended in a final concentration of 10^6^ cells/ml in hypotonic propidium iodide (PI) solution containing 0.1% Sodium citrate, 0.3% Trixon-X 100, 0.01% PI and 0.002% Ribonuclease A. Cells were incubated in 4°C for 30 min and analyzed on a FACScan (Becton-Dickinson, Franklin Lakes, NJ) using the software CELLQuest. One hundred thousand cells were collected for each sample. Excitation occurred at 488 nm and data was collected using the FL2 channel and analyzed using FCS ExpressV3.

### Western Blot Analysis

Confluent cultures of PANC-1 or Mia PaCa-2 cells grown on 3.5 cm dishes were washed and then incubated for 24 h with DMEM containing 5 mM glucose and 1% FBS. The cells were then washed twice with DMEM containing 5 mM glucose and incubated in serum-free medium in the absence or presence of berberine, metformin or A-769662, as described in the individual experiments. The cultures were then directly lysed in 2× SDS-PAGE sample buffer [200 mM Tris-HCl (pH 6.8), 2 mM EDTA, 0.1 M Na_3_VO_4_, 6% SDS, 10% glycerol, and 4% 2-mercaptoethanol], followed by SDS-PAGE on 10% gels and transfer to Immobilon-P membranes (Millipore, Billerica, MA). Western blots were then performed on membranes incubated overnight with the specified antibodies in phosphate-buffered saline (PBS) containing 0.1% Tween-20. The immunoreactive bands were detected with ECL (enhanced chemiluminescence) reagents (GE Healthcare Bio-Sciences Corp, Piscataway, NJ). The antibodies used detected the phosphorylated state of S6K at Thr^389^, S6 at Ser^240/244^ and ERK1/2 at Thr^202^ and Tyr^204^, AMPKα at Thr^172^, ACC at Ser^79 and^ Raptor at Ser^792^. In addition, the total level of these proteins was also evaluated. Routinely, the membranes used with the phospho-specific antibodies were stripped and re-blotted with the antibodies that detect the total level of the corresponding protein. Occasionally, stripping and re-blotting of the same membrane was not satisfactory for obtaining both loading and expression controls. In these cases, we used a separate blot for assessing total protein expression and immunoblotted the original membrane for other proteins (e.g. actin, S6K, S6, ERK) that migrate at a different position in the original gel for verifying equal loading.

### Cell Proliferation

Cultures of PANC-1 and MiaPaCa-2 cells, 3–5 days after passage, were washed and suspended in DMEM containing 5 mM glucose. Cells were then disaggregated by two passes through a 19-guage needle into an essentially single-cell suspension as judged by microscopy. Cell number was determined using a Coulter Counter, and 2×10^4^ cells were seeded in 35 mm tissue culture plates in DMEM containing 5 mM glucose and 10% FBS. After 24 h of incubation, the medium was removed and the cultures shifted to DMEM containing 5 mM glucose without or with 3% FBS. The cultures were then incubated in a humidified atmosphere containing 10% CO_2_ at 37°C for 4 days and the total cell count was determined from a minimum of four dishes per condition using a Coulter counter, after cell clumps were disaggregated by passing the cell suspension ten times through a 19- and subsequently a 21-gauge needle.

### Mice xenografts

Early-passage MiaPaCa-2 cells were harvested, and 2×10^6^ cells were implanted into the right flanks of male *nu/nu* mice. The male *nu/nu* mice were maintained in specific pathogen-free facility at University of California at Los Angeles (UCLA). The UCLA Chancellor’s Animal Research Committee approved all the animal experiments. The animals were randomized into control and treated groups (10 mice per group) and were given punched ear tags to allow identification. Treatment was initiated when the tumors reached a mean diameter of 2 mm, and the 1st day of treatment in both cases was designated as day 0. For injection into animals, metformin (250 mg/Kg), berberine (5 mg/kg) or vehicle (control) was given intraperitoneally once daily intraperitoneally (50 µL/mouse). Tumor volume (*V*) was measured by external caliper every 4 days and it was calculated as *V* = 0.52 (length × width^2^). At the end of the experiment, the tumors were dissected weighted and measured. The volume of the excised tumors was calculated as *V* = 0.52 (length × width × depth).

### Statistical analysis

Values are means ± SE. Differences between groups were analyzed with the unpaired Student’s *t*-test.

## Results

### Berberine inhibits DNA synthesis, cell cycle progression and cell proliferation in PDAC cells

**Figure 1:**
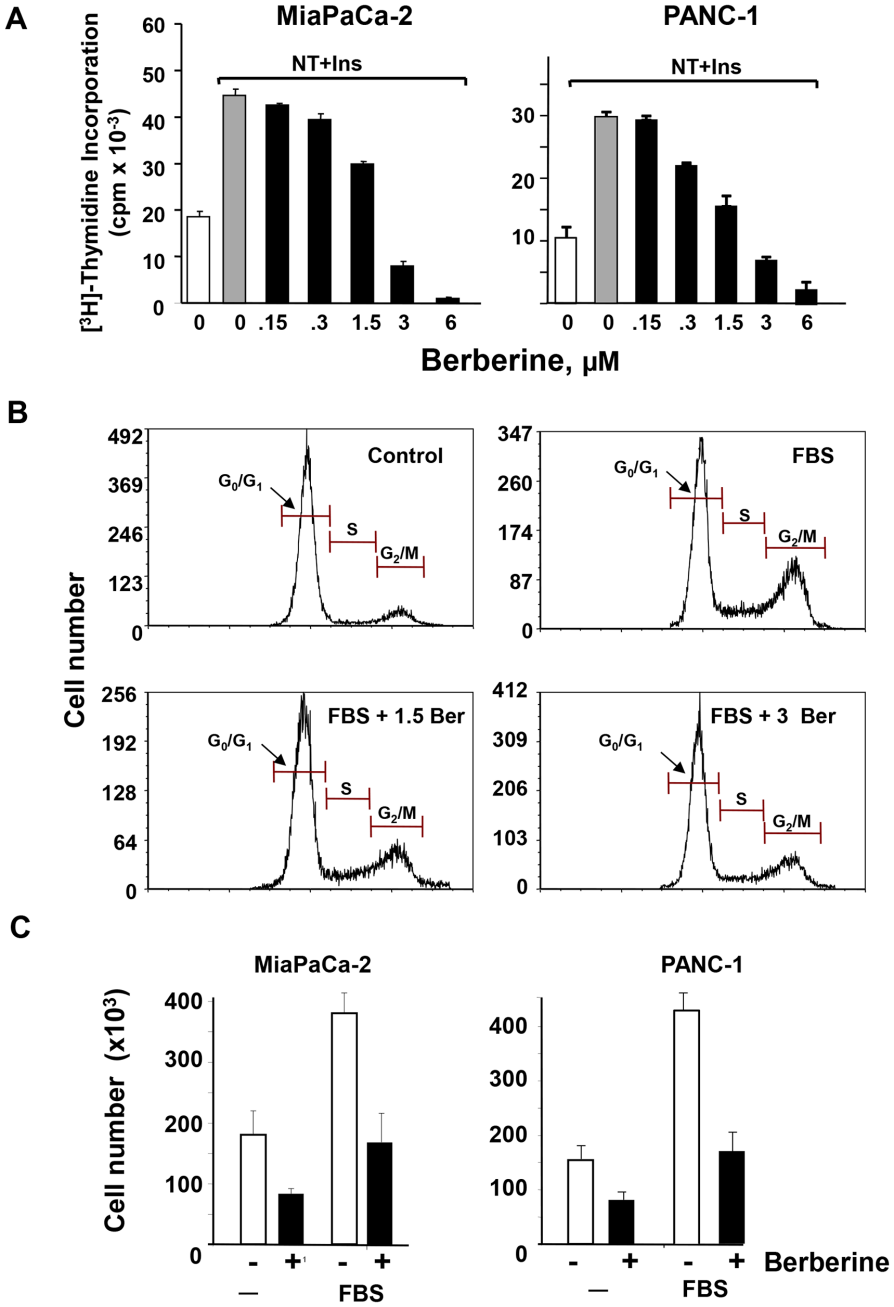
Berberine inhibits DNA synthesis, cell cycle progression and proliferation in PANC-1 and Mia PaCa-2 cells. **A,** Mia PaCa-2 or PANC-1 cells were incubated without (*open bars*) or with 5 nM neurotensin and 10 ng/ml insulin (*closed bars*) in the presence of increasing concentration of berberine for 17 h at 37°C prior to the addition of [^3^H]-thymidine for 6 h. The radioactivity incorporated into acid-insoluble pools was measured in a scintillation counter, as described in “Materials and Methods. The values shown are the mean ± SEM obtained in 3 independent experiments; **B,** PANC-1 cells were treated without (control) or with berberine at 1.5 µM or 3 µM in medium containing 2.5% FBS for 3 days (indicated by cont., FBS, FBS +1.5 Ber and FBS +3 Ber). Cell cycle was analyzed by PI-staining and flow cytometry. Similar results were obtained in 3 independent experiments. **C,** Single-cell suspension of Mia PaCa-2 or PANC-1 cells were plated on tissue culture dishes at a density of 2×10^4^ cells per dish. After 24 h of incubation the medium was removed and the cultures shifted to medium without or with 3% FBS in the absence (open bars) or presence (closed bars) of 3 µM berberine. The cultures were incubated for 4 days as described in “Materials and Methods”. Cell count was determined from 4 to 6 replicate plates per condition using a Coulter Counter. Results are presented as mean ± SEM of 3 biological replicates.

Initially, we determined the effect of the isoquinoline alkaloid berberine on DNA synthesis in PANC-1 and MiaPaCa-2 cells, which have been used extensively as models of PDAC cells. Cultures of these cells grown in medium containing 10% fetal bovine serum were washed and transferred to serum-free medium for 24 h. Then, the cells were switched to medium containing a physiological concentration of glucose (5 mM), increasing doses of berberine and a combination of insulin (10 ng/ml) and the GPCR agonist neurotensin (5 nM) to elicit potent mitogenic crosstalk signaling [20], [21], [46]. Treatment with berberine inhibited the stimulation of DNA synthesis in a dose-dependent manner in both PANC-1 and MiaPaCa-2 cells. At a concentration of 3 µM, berberine inhibited DNA synthesis by 82% in MiaPaCa-2 cells and by 76% in PANC-1 cells. The incorporation of [^3^H]-thymidine was blocked by 97% in MiaPaCa-2 cells and by 94% in PANC-1 cells in response to 6 µM berberine (**Fig. 1 A**).

The assays of [^3^H]-thymidine incorporation were complemented by flow cytometric analysis to determine the proportion of PDAC cells in the various phases of the cell cycle. As shown in **Fig. 1B** **,** exposure of PANC-1 cells to berberine (1.5–3 µM) induced a marked increase in the proportion of cells in G_1_ (from 61±0.2% in cells with FBS to 79±2% in cells with FBS and berberine) and a corresponding decrease in the proportion of cells that were in S and G_2_/M phase of the cell cycle (from 36±0.1% to 19±0.7%). These results indicate that berberine delays the progression of the PDAC cell cycle at G_1_.

We next examined the effect of berberine on the proliferation of pancreatic cancer cells. Single cell suspensions of either PANC-1 or MiaPaCa-2 cells were plated and incubated in media supplemented without or with 3% FBS in the absence or presence of 3 µM berberine. Treatment with berberine markedly inhibited proliferation in both PDAC cells (**Fig. 1C**) without affecting cell viability at the doses tested (results not shown). Taken together, the results in **Fig. 1** demonstrate that berberine inhibits DNA synthesis, cell cycle progression and proliferation in pancreatic cancer cells.

### Berberine and metformin inhibit the growth of a PDAC xenograft in nude mice

**Figure 2:**
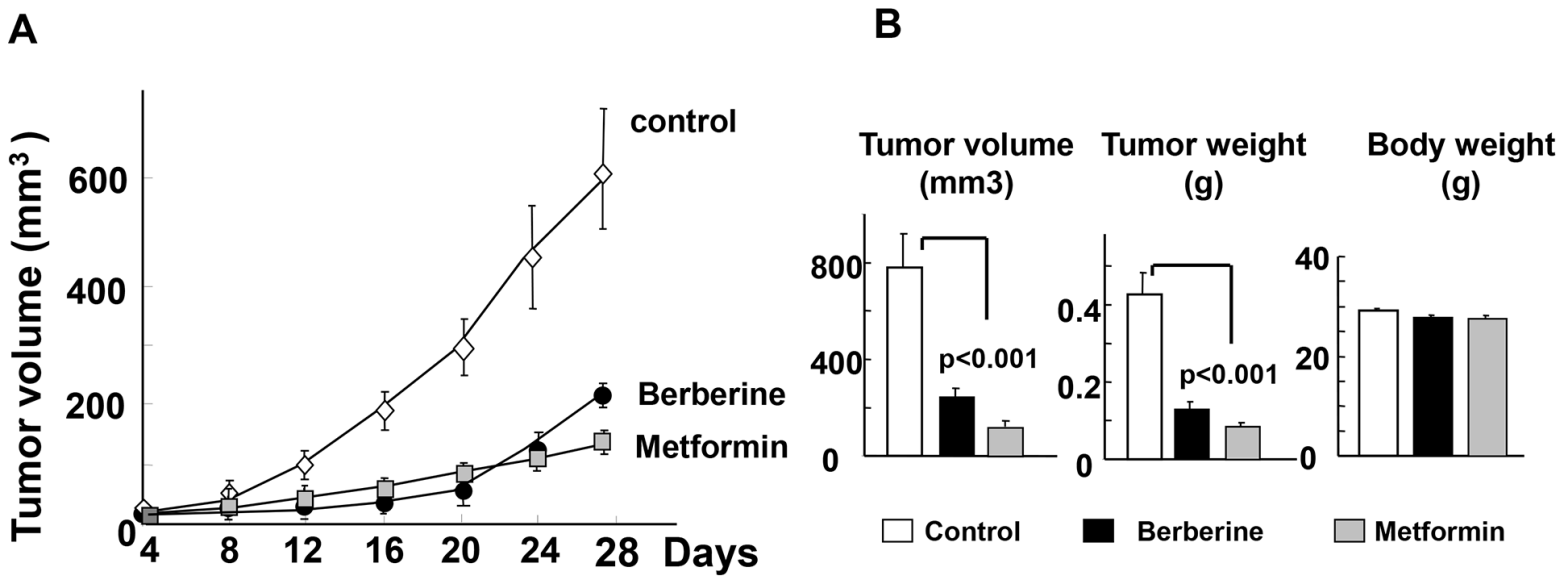
Berberine inhibits the growth of MiaPaCa-2 tumor xenografts as effectively as metformin. Xenografts of MiaPaca-2 were generated by implantation of 2×10^6^ cells into the right flanks of male *nu/nu* mice. When the tumors reached a mean diameter of 2 mm the animals were randomized into control and treated groups (10 mice per group). Berberine was given once daily intraperitoneally at 5 mg/kg for the duration of the experiment. For comparison, metformin was given intraperitoneally to another group of mice at 250 mg/kg. The 1^st^ day of treatment was designated as day 0. Control animals received an equivalent volume of saline. **A**, Tumor volumes were measured every 4 days as described in “Materials and Methods”. At the end of the experiment (day 29, the tumors were removed, weighted and measured and tumor volumes estimated as *V* = 0.52 (length × width × depth). The results are shown in panel **B** (mean ± SEM). Treatment of mice with berberine significantly reduced the volume and weight of the tumors as compared with the tumors from the control group (p<0.001), as indicated. A similar inhibition of tumor growth was obtained by administration of metformin (p<0.001). The curves corresponding to the growth of MiaPaca-2 xenografts in mice treated with berberine or metformin were superimposable during the first 24 days. At day 29, metformin was slightly more effective than berberine as assessed by tumor volume (p<0.05) but the difference of the effects between these drugs did not reach statistical significance (p>0.07) when scored by tumor weight (**Fig. 2, B**). At the concentrations used, berberine and metformin were well tolerated with no apparent toxicity based on body weight changes.

Given our results showing inhibitory effects of berberine on PDAC cell proliferation *in vitro*, we subsequently determined whether this compound inhibits pancreatic cancer growth *in vivo* using MiaPaca-2 tumor xenografts in nude mice. The xenografts were derived by implantation of 2×10^6^ cells into the right flanks of male *nu/nu* mice. The animals were randomized into control and berberine-treated groups (10 mice per group). Berberine was given once daily intraperitoneally at 5 mg/kg for the duration of the experiment. As shown in **Fig. 2** **,** administration of berberine decreased the growth of MiaPaca-2 cells xenografted in nude mice by 70%. The tumor volumes at the end of the experiment (day 29) were 781 mm^3^ in the control and 240 mm^3^ in the berberine treated group (p<0.001). The dose of berberine used (5 mg/kg) is 12-fold lower than the LD_50_, based on preliminary range-finding studies and previous work published by others [53], [68], [69]. Berberine was well tolerated and did not significantly affect the weight of the mice during the treatment (**Fig. 2, B**). The inhibitory effect of berberine was comparable to that induced by metformin given at 250 mg/Kg (**Fig. 2**), a dose that induces maximal inhibitory effect on PDAC tumor growth [70]. Indeed, the curves corresponding to the growth of MiaPaca-2 xenografts in mice treated with berberine or metformin were virtually superimposable during the first 24 days (**Fig. 2, A**). At day 29, metformin was slightly more effective than berberine as assessed by tumor volume (p<0.05) but the effects did not reach statistical significance (p>0.07) when scored by tumor weight (**Fig. 2, B**). These results indicate that berberine inhibits the growth of human pancreatic cancer cells xenografted into nude mice with efficacy comparable to that achieved by a maximal dose of metformin.

### Berberine induces mitochondrial membrane depolarization, reduces the levels of ATP and stimulates AMPK in pancreatic cancer cells

**Figure 3:**
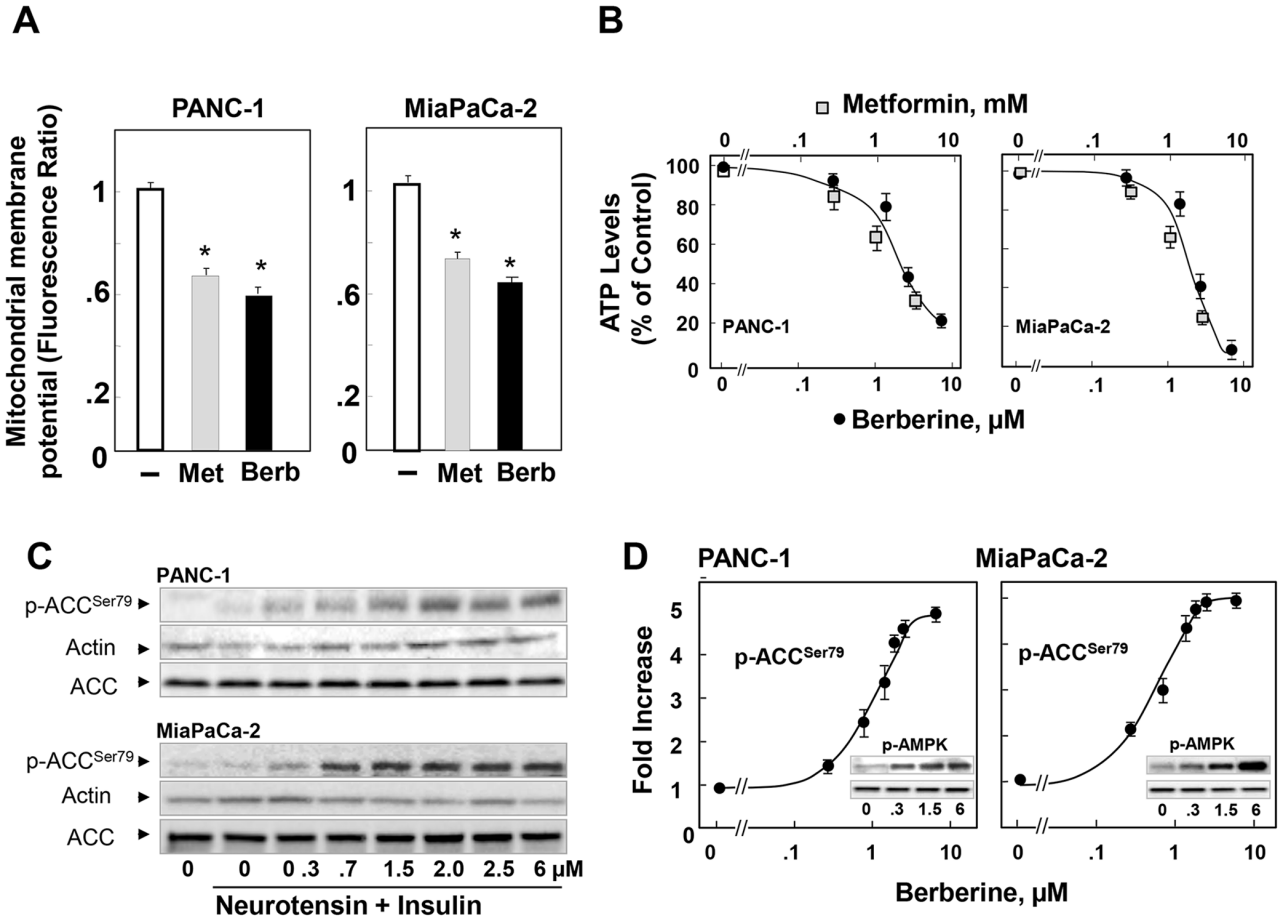
Berberine and metformin induce mitochondrial membrane depolarization, reduce ATP levels and activate AMPK in PDAC cells. **A,** Cultures of PANC-1 and MiaPaca-2 cells were incubated in the absence or in the presence of 3 µM berberine (Berb) or 1 mM metformin (Met) for 17 h in DMEM containing 5 mM glucose. The change in mitochondrial membrane potential was measured using the mitochondrial membrane potential indicator JC-1. The results are expressed as an average ratio of red/green florescent intensity in a single visual field (mean ± SEM). At least 5 fields were studied in each condition. P values were determined using the t-test (SigmaPlot 12.); *p<0.002. **B,** Cultures of PANC-1 and MiaPaca-2 cells were incubated in the absence or in the presence of berberine or metformin at the indicated concentrations for 17 h in DMEM containing 5 mM glucose and 2.5% FBS. **C,** Cultures of PANC-1 (upper panels) and MiaPaCa-2 (lower panels) were incubated in the absence or in the presence of berberine at the indicated doses for 17 h. Then, the cells were stimulated for 1 h with 5 nM neurotensin and 10 ng/ml insulin and lysed with 2X SDS-PAGE sample buffer. The samples were analyzed by SDS-PAGE and immunoblotting with antibodies that detect the phosphorylated state of Acetyl-CoA Carboxylase (ACC) at Ser^79^. Western blotting for actin was used to verify equal loading in the same membrane and a separate gel confirmed that expression of total ACC protein was not changed by any of the treatments. **D,** Quantification was performed using Multi Gauge V3.0. The values represent the mean ± SEM; n = 3, fold increase in ACC phosphorylation at Ser^79^. Inset, phosphorylated state of AMPK at Thr^172^ at the indicated concentrations of berberine (µM).

Having established that berberine inhibits PDAC cell proliferation *in vitro* and *in vivo*, we next explored the mechanisms involved. In other cell types, berberine is thought to inhibit complex I of the mitochondrial respiratory chain [71], [72], resulting in reduced ATP synthesis and concomitant increase in cellular AMP and ADP which are potent allosteric activators of AMPK [73]–[75]. Because it is not known whether berberine has any effect on mitochondrial function in pancreatic cancer cells, we initially examined whether this phytochemical interferes with mitochondrial membrane potential in these cells. Cultures of MiaPaCa-2 and PANC-1 cells were incubated in the absence or presence of 3 µM berberine or 1 mM metformin, included for comparison. Then, mitochondrial membrane potential, a key component driving ATP synthesis, was assessed using the mitochondrial-specific fluorescent probe JC-1 [76]. As shown in **Fig. 3 A,** berberine caused a significant fall in mitochondrial membrane potential in MiaPaCa-2 and PANC-1 cells. The effect was comparable to that induced by metformin (**Fig. 3 A**). These results indicate that berberine, like metformin, targets mitochondrial function in PDAC cells. Accordingly, exposure to increasing concentrations of berberine or metformin produced a marked dose-dependent decrease in the intracellular ATP levels in both PANC-1 and MiaPaCa- 2 cells (**Fig. 3 B**).

We next determined whether berberine stimulates AMPK activity within intact MiaPaCa-2 and PANC-1 cells. Lysates of these cells were analyzed by immunoblotting using antibodies that detect the phosphorylated state of acetyl-CoA carboxylase (ACC) at Ser^79^, a residue directly phosphorylated by AMPK and used as a biomarker of AMPK activity within intact cells [75]. Treatment with berberine induced a marked increase in the phosphorylation of ACC at Ser^79^ in a dose-dependent manner in both PANC-1 and MiaPaCa-2 cells (**Fig. 3, C** **;** quantification in **Fig. 3 D**). Maximal effect was elicited at doses >2 µM in both PDAC cells (**Fig. 3 D**). Furthermore, treatment of MiaPaCa-2 or PANC-1 cells with berberine induced a striking increase in the phosphorylation of AMPK at Thr^172^, the residue in the kinase domain of the catalytic subunit (α) of AMPK critical for activation (Inserts in **Fig. 3 D**). Collectively, the results demonstrate that berberine decreases mitochondrial membrane potential and lowers intracellular levels of ATP thereby stimulating AMPK activity in PDAC cells cultured in medium containing a physiological concentration of glucose (5 mM).

### Berberine inhibits mTORC1 and ERK activation in PDAC cells

**Figure 4:**
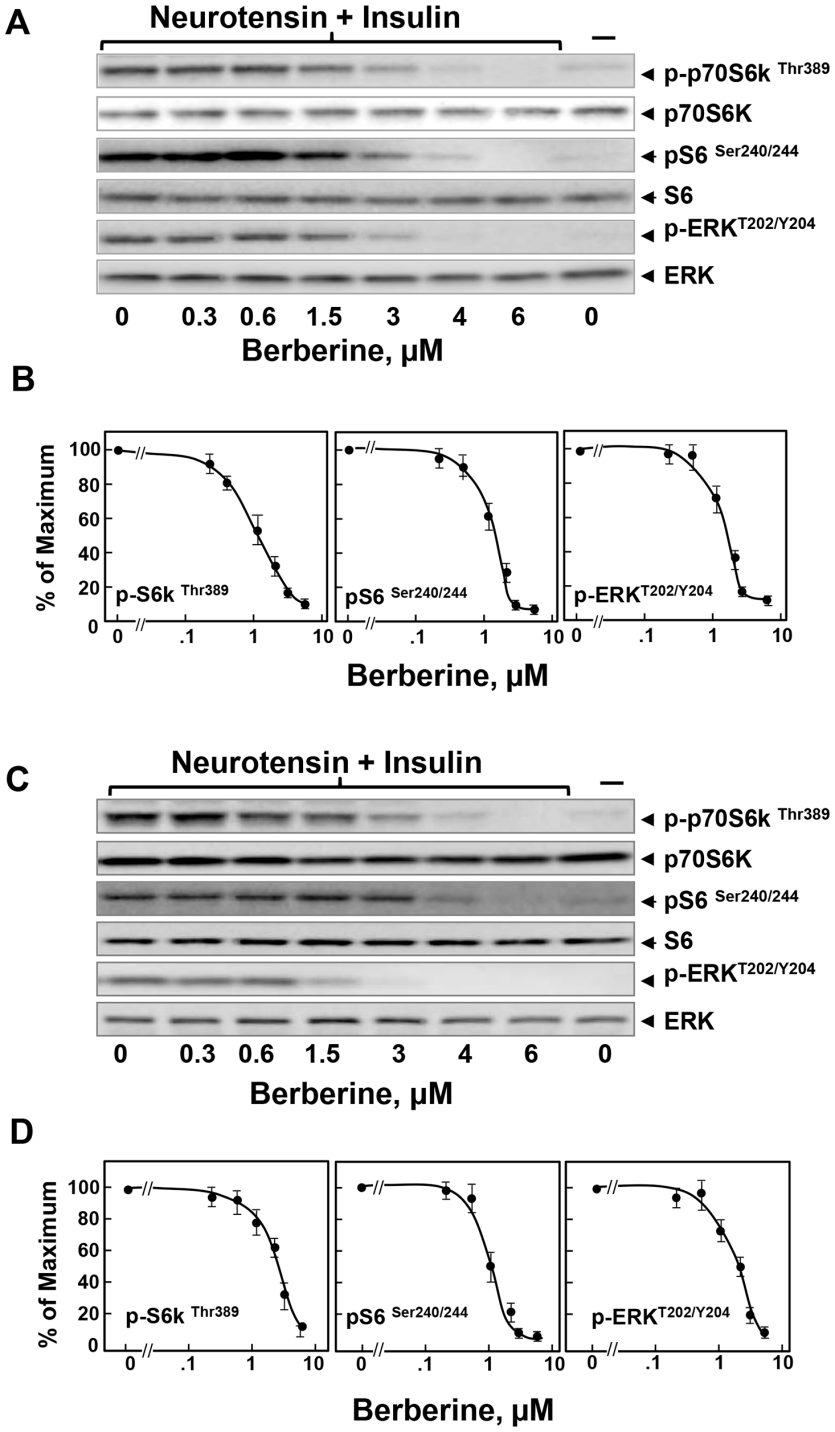
Berberine inhibits mTORC1 signaling and ERK activation in PDAC cells. Cultures of MiaPaCa-2 (Panels **A** and **B**) or PANC-1 cells (panels **C** and **D**) were incubated in the absence or in the presence of increasing concentrations of berberine. Then, the cells were stimulated for 1 h with 5 nM neurotensin and 10 ng/ml insulin and lysed with 2X SDS-PAGE sample buffer. The samples were analyzed by SDS-PAGE and immunoblotting with antibodies that detect the phosphorylated state of S6K at Thr^389^, S6 at Ser^240/244^, and ERK at Thr^202^ and Tyr^204^. Immunoblotting with total S6K, S6 and ERK was used to verify equal gel loading. The quantification of the immune signals was performed using Multi Gauge V3.0. The results are presented in the plots shown in panels **B** and **D.** The values represent the mean ± SEM (n = 3) of S6K, S6 and ERK phosphorylation expressed as a percentage of the maximal response obtained in 3 independent experiments.

The activation of the PI3K/Akt/mTORC1 and MEK/ERK pathways plays a pivotal role in stimulating DNA synthesis, cell cycle progression and proliferation of PDAC cells and are negatively regulated by AMPK [21]. Consequently, we determined whether berberine inhibits mTORC1 and ERK activation in PDAC cells. Cultures of MiaPaCa-2 cells were treated with increasing doses of berberine and then stimulated with a combination of insulin and neurotensin to elicit positive crosstalk (**Fig. 4**). Lysates of these cells were analyzed by immunoblotting using antibodies that detect the phosphorylated state of S6K at Thr^389^, a residue directly phosphorylated by mTORC1, using Western blot analysis with antibodies that specifically detect the phosphorylated state of this residue. To corroborate that phosphorylation of S6K at Thr^389^ reflects its activation within PDAC cells, we examined the phosphorylation of the 40S ribosomal protein subunit S6, a downstream target of S6K [77]. As shown in **Fig. 4 A** **,** stimulation with insulin and neurotensin induced a marked increase in mTORC1 activity, as scored by phosphorylation of S6K and S6 protein (pS6K and pS6). Treatment with berberine prevented mTORC1 activation in a dose-dependent manner (**Fig. 4A**; quantification in **Fig. 4 B**).

Given the pivotal importance of the RAS/MEK/ERK pathway in PDAC development and maintenance, we also analyzed the effect of increasing concentrations of berberine on ERK activation by detecting ERK phosphorylated on Thr^202^ and Tyr^204^. The results in **Fig. 4 A, B** demonstrated that berberine prevented ERK activation in MiaPaCa-2 cells, in a dose-dependent manner. The doses of berberine that inhibited ERK activation were similar to those that blunted mTORC1 activation and produced AMPK activation.

Similar to the results obtained with MiaPaCa-2 cells, berberine inhibited mTORC1 and ERK activation by insulin and neurotensin in a dose-dependent manner in PANC-1 cells (**Fig. 4 C** **;** quantification in **Fig. 4 D**). We also found that berberine inhibited mTORC1 and ERK signaling in PANC-1 cells stimulated with FBS instead of insulin and neurotensin (**S1 Figure**). The results presented so far demonstrate that berberine inhibited mTORC1, ERK and cell proliferation in PDAC cells at doses that reduced the intracellular ATP levels and induced robust AMPK activation.

### Knockdown of the α subunits of AMPK reverses inhibition of mTORC1, ERK and DNA synthesis induced by low doses of berberine or metformin: evidence for AMPK-dependent and AMPK-independent mechanisms

**Figure 5:**
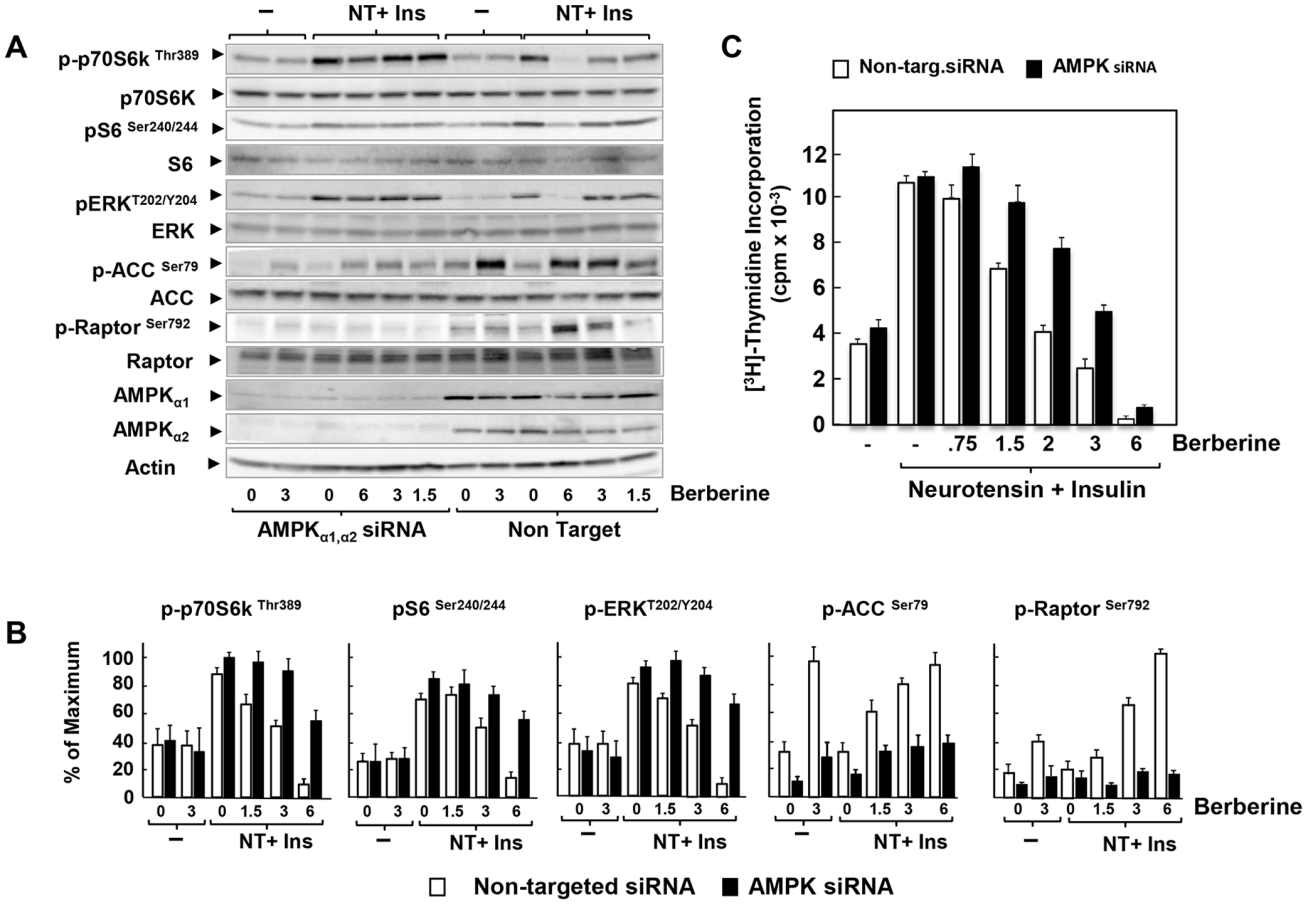
Knockdown of the α subunits of AMPK reverses inhibition of mTORC1, ERK and DNA synthesis induced by low but not high doses of berberine. **A**, PANC-1 cells were transfected with either non-targeting negative control (Non Target.) or 10 nM AMPKα1 and 10 nM AMPKα2 siRNA (AMPKα1, α2 siRNA) in DMEM containing 5 mM glucose and 10% FBS. After 3 days the cells were incubated either in the absence or presence of berberine for 17 h in serum free DMEM containing 5 mM glucose. Then, the cells were stimulated for 1 h with 5 nM neurotensin and 10 ng/ml insulin and lysed with 2X SDS-PAGE sample buffer. The samples were analyzed by SDS-PAGE and immunoblotting with the following phospho antibodies: S6K at Thr^389^, S6 at Ser^240/244^, and ERK at Thr^202^ and Tyr^204^, ACC at Ser^79^ and Raptor at Ser^792^. Shown here is a representative autoluminogram; similar results were obtained in 4 independent experiments. **B,** Quantification was performed using Multi Gauge V3.0. Results are expressed as the percentage of the maximum (mean ±SEM: n = 4). **C,** PANC-1 cells were transfected with either non-targeting negative control (open bars) or 10 nM AMPKα1 and 10 nM AMPKα2 siRNA (black bars) in DMEM containing 5 mM glucose and 10% FBS. After 3 days the cells were incubated for 6 h in serum-free medium containing 5 mM glucose. Then, 5 nM neurotensin and 10 ng/ml insulin and the indicated concentration of berberine were added for 17 h at 37°C prior to the addition of [^3^H]-thymidine for 6 h. The radioactivity incorporated into acid-insoluble pools was measured in a scintillation counter, as described in “Materials and Methods”. Results are expressed as the percentage of maximum mean ± SEM obtained in 4 independent experiments (3 replicate cultures per point in each experiment).

**Figure 6:**
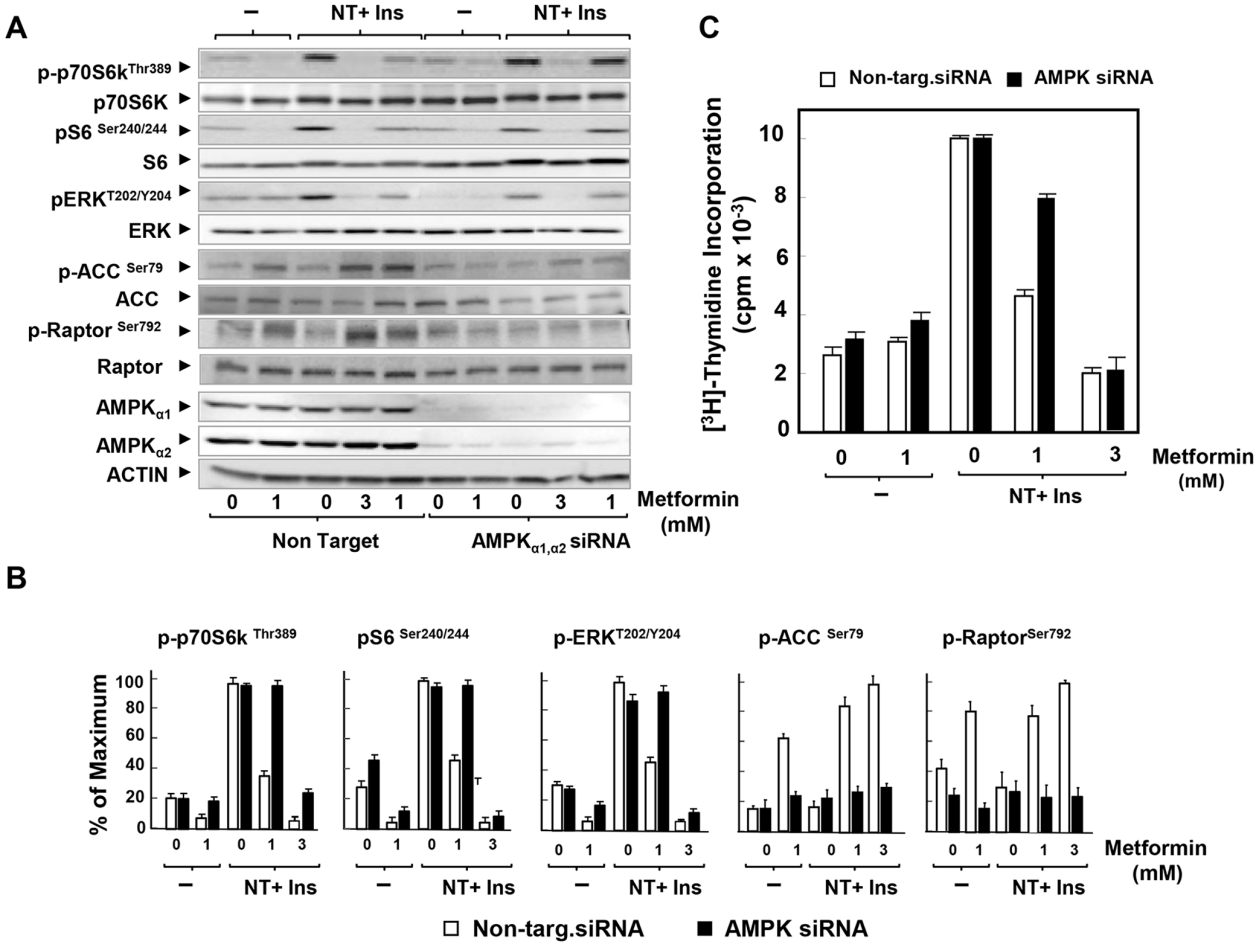
Knockdown of the α subunits of AMPK reverses inhibition of mTORC1, ERK and DNA synthesis induced by low doses of metformin. **A,** PANC-1 cells were transfected with either non-targeting negative control (Non Target.) or 10 nM AMPKα1 and 10 nM AMPKα2 siRNA (AMPKα1, α2 siRNA) in DMEM containing 5 mM glucose and 10% FBS. After 3 days the cells were incubated either in the absence or presence of 1 mM or 3 mM metformin (as indicated) for 17 h in serum free DMEM containing 5 mM glucose. The samples were analyzed by SDS-PAGE and immunoblotting with the following phospho antibodies: S6K at Thr^389^, S6 at Ser^240/244^, ERK at Thr^202^ and Tyr^204^, ACC at Ser^79^ and Raptor at Ser^792^ Shown here is a representative autoluminogram; similar results were obtained in 3 independent experiments. **B,** Quantification was performed using Multi Gauge V3.0. Results are expressed as the percentage of maximum (mean ±SEM; n = 3). **C,** PANC-1 cells were transfected with either non-targeting negative control (open bars) or 10 nM AMPKα1 and 10 nM AMPKα2 siRNA (black bars) in DMEM containing 3 mM glucose and 10% FBS. After 3 days the cells were incubated for 6 h in serum-free medium containing 5 mM glucose. Then, 5 nM neurotensin and 10 ng/ml insulin and metformin at either 1 mM or 3 mM were added for 17 h at 37°C prior to the addition of [^3^H]-thymidine for 6 h. The radioactivity incorporated into acid-insoluble pools was measured in a scintillation counter, as described in “Materials and Methods”. Results are expressed as the percentage of maximum mean ±SEM obtained in 3 independent experiments (3 replicate cultures per point in each experiment).

In order to determine the role of AMPK in mediating berberine-induced inhibition of PDAC cell signaling and proliferation, we used short interfering RNA (siRNA), which effectively (>90%) knockdown the protein expression of both α_1_ and α_2_ catalytic subunits of AMPK, as compared with cells transfected with non-targeting siRNA (**Fig. 5, A**). Accordingly, berberine-induced increases in the phosphorylation of ACC at Ser^79^ and Raptor at Ser^792^, were blunted in the cells treated with siRNA targeting the α_1_ and α_2_ catalytic subunits of AMPK (**Fig. 5, A** **;** quantification in **Fig. 5, B**). The salient feature in **Fig. 5, A** is that knockdown of AMPK prevented the inhibitory effect produced by treatment with low doses of berberine (<3 µM) on the stimulation of mTORC1 (scored by phosphorylation of S6K at Thr^389^ and of its substrate S6) and ERK activation in PDAC cells. In contrast, knockdown of AMPK expression prevented only partially the inhibitory effect berberine added at 6 µM. It was conceivable that the inhibitory effect of berberine at the high concentration was due to incomplete elimination of AMPK after silencing. However, the dose-response relationships presented in **Figs. 2** and **5** argue against this possibility. Specifically, ACC phosphorylation at Ser^79^ reached a plateau in cells challenged with berberine at doses 2–6 µM (**Fig. 2**) or 3–6 µM (**Fig. 5B**). The faint ACC phosphorylation remaining after knockdown of AMPK followed a similar dose-response relationship, i.e. phosphorylation was increased only slightly and to same level by either 3 µM or 6 µM berberine in AMPK-depleted cells (**Fig. 5**). Furthermore, the marked increase in Raptor phosphorylation at Ser^792^ in response to berberine at 6 µM was also blocked by knockdown of AMPK expression (**Fig. 5A**; quantification in **Fig. 5, B**). Thus, knockdown of AMPK prevented berberine-induced phosphorylation of the AMPK substrates ACC at Ser^79^ and Raptor at Ser^792^ when added at either at 3 µM or 6 µM. Consequently, berberine hampered mTORC1 and ERK activation through AMPK signaling at low doses but at higher concentrations, berberine inhibited mitogenic signaling, at least in part, through an AMPK-independent pathway in PDAC cells.

In accord with this conclusion, knockdown of α_1_ and α_2_ catalytic subunit expression of AMPK substantially prevented the inhibitory effect produced by low doses of berberine on the stimulation of DNA synthesis in PDAC cells (**Fig. 5, C**). In contrast, at higher concentrations, berberine inhibited DNA synthesis through an AMPK-independent mechanism. These results reinforce the notion that berberine inhibits mitogenic signaling in PDAC cells through distinct AMPK-dependent and independent mechanisms in a dose-dependent manner.

Previously, we demonstrated that metformin, at low concentrations, inhibited DNA synthesis through an AMPK-dependent mechanism in PANC-1 cells incubated in medium containing physiological concentrations of glucose [44]. The results obtained here with berberine prompted us to examine further the notion that inhibitors of mitochondrial function impede mitogenic signaling through AMPK-dependent and independent mechanisms in a dose-dependent manner. Knockdown of α_1_ and α_2_ catalytic subunit expression of AMPK prevented the increase in the phosphorylation of ACC at Ser^79^ and Raptor at Ser^792^ in cells treated with metformin at either 1 mM or 3 mM (**Fig. 6 A**; quantification in **Fig. 6, B**). Interestingly, siRNA-mediated depletion of AMPK reversed the inhibitory effect produced by 1 mM metformin on mTORC1 (scored by phosphorylation of S6K at Thr^389^ and of its substrate S6) and ERK activation in PDAC cells (**Fig. 6 A**; quantification in **Fig. 6, B**). In contrast, knockdown of AMPK did not prevent the inhibitory effect of metformin on mTORC1 and ERK when added at 3 mM (**Fig. 6A** ; quantification in **Fig. 6, B**). Furthermore, knockdown of α_1_ and α_2_ catalytic subunit expression of AMPK substantially reversed the inhibitory effect produced by 1 mM metformin on the stimulation of DNA synthesis in PDAC cells but did not prevent inhibition of DNA synthesis produced by 3 mM metformin. Collectively, these results support the notion that structurally unrelated inhibitors of mitochondrial function, including berberine and metformin, inhibit mitogenic signaling in PDAC cells through AMPK-dependent and independent mechanisms in a dose-dependent manner.

To substantiate the operation of AMPK-mediated inhibition of mitogenic signaling in pancreatic cancer cells, we determined whether treatment with A-769662, a direct AMPK agonist [78], [79], inhibits mTORC1 and DNA synthesis in PANC-1 cells. As expected for a compound that acts directly on AMPK rather than through inhibition of mitochondrial function, A-769662 neither reduced mitochondrial membrane potential nor decreased ATP levels in PANC-1 cells but induced robust phosphorylation of ACC at Ser^79^ and Raptor at Ser^792^ (**S2 Figure**). At the concentrations used, A-769662 inhibited mTORC1-mediated phosphorylation of S6K at Thr^389^ and of S6 at Ser^240/244^ and DNA synthesis in PANC-1 cells (**S2 Figure**). The results corroborate that AMPK activation inhibits mitogenic signaling in pancreatic cancer cells.

## Discussion

The studies presented here were designed to explore the hypothesis that structurally unrelated natural or synthetic compounds that interfere with mitochondrial-mediated ATP synthesis and target mTORC1 and ERK pathways, provide novel anti-PDAC agents. Our results demonstrate that treatment of pancreatic cancer PANC-1 and MiaPaCa-2 cells with berberine potently inhibited DNA synthesis, cell cycle progression and proliferation in a dose-dependent manner. We noticed that most previous studies examining effects of berberine *in* *vitro* were carried out with cancer cells cultured in medium supplemented with supra-physiological concentrations of glucose (e.g. 25 mM, as in DMEM) and used berberine at doses as high as 50 µM [61], [80], [81]. When PDAC cells were cultured in medium containing a physiological concentration of glucose, as in this study, berberine induced growth-suppressive effects at a dose as low as 1.5–3 µM. In view of the inhibitory effects of berberine on the proliferative responses of PDAC cells *in vitro*, we examined whether this compound inhibits pancreatic cancer growth using the MiaPaca-2 tumor xenograft model in nude mice. Our results show that administration of berberine markedly inhibited the growth of human pancreatic cancer cells xenografted into nude mice, as effectively as metformin. Given that berberine inhibited PDAC cell proliferation both *in vitro* and *in vivo,* it was important to elucidate its mechanism of inhibitory action in these cells.

Berberine has been proposed to inhibit complex I of the mitochondrial respiratory chain, reduce ATP synthesis and thereby activate AMPK, a highly conserved sensor of cellular energy being activated when ATP concentrations decrease and 5′-AMP concentrations increase [73]. Accordingly, we demonstrate here that exposure of PDAC cells to berberine decreased mitochondrial membrane potential and induced a marked, dose-dependent decline in the intracellular levels of ATP. Concomitantly, berberine produced a pronounced dose-dependent stimulation of AMPK, as judged by the increase in the phosphorylation of ACC at Ser^79^, a reliable biomarker of AMPK activity within intact cells [75] and phosphorylation Raptor at Ser^792^. AMPK has been proposed to inhibit mTORC1 activation by phosphorylation of TSC2 [40]–[42], Raptor [82] and IRS-1 [83], [84]. Accordingly, we demonstrate here that berberine inhibited mTORC1 activity in PDAC cells, as shown by monitoring the phosphorylated state of S6K at Thr^389^, a residue directly phosphorylated by mTORC1 and the phosphorylation of S6, a downstream target of S6K [77]. Furthermore, berberine also inhibited ERK activation in PDAC cells. The inhibitory effects of berberine on mTORC1 and ERK were elicited at doses that hampered mitochondrial function, reduced intracellular levels of ATP and activated AMPK within intact PDAC cells.

Although the preceding results are consistent with the notion that AMPK mediates some of the inhibitory effects of berberine on mitogenic signaling in PDAC cell, the precise role of AMPK in the proliferation and survival of cancer cells has become controversial [85]. Specifically, it remains unclear whether AMPK suppresses cancer cell proliferation (tumor suppressive function) or alternatively enhances cancer cell survival under conditions of metabolic stress (tumor promoter function). A tumor suppressive activity of AMPK is strongly implicated in Myc-induced lymphomagenesis [86], aerobic glycolysis [86] and in the mechanism underlying the gain of oncogenic function of certain p53 protein mutants [87]. Conversely, several recent reports have also shown that AMPK promotes tumorigenesis via protecting cancer cell viability under energy stress conditions [88] and enhances metabolic transformation [89]. These contrasting views prompted us to determine the role of AMPK in human pancreatic cancer cells. Specifically, we examined whether knockdown of the protein expression of both α_1_ and α_2_ catalytic subunits of AMPK in these cells opposes or facilitates the inhibitory effects induced by berberine and metformin, two agents that induce metabolic stress via inhibition of mitochondrial function and interference with ATP synthesis.

Our results led us to propose a novel mechanism of action for these agents that is sharply dependent on the dose used. We found that knockdown of the α subunits of AMPK reversed the inhibition of mTORC1 and ERK induced by low doses of berberine. Consequently, we propose that berberine inhibits mitogenic signaling through an AMPK-dependent pathway when used at low concentrations and in PDAC cells cultured in physiological concentration of ambient glucose. However, at higher concentrations, berberine inhibited mitogenic signaling (mTORC1 and ERK) and DNA synthesis through an AMPK-independent mechanism. Importantly, AMPK knockdown prevented the increase in the phosphorylation of ACC at Ser^79^ and Raptor at Ser^792^ induced by berberine at either low or high doses.

A number of studies, using high concentrations of metformin (e.g. 10 mM, as in [39], [90]) indicated that the inhibitory effect of metformin on mTORC1 is not dependent on AMPK but the significance of results obtained with metformin at such high doses has been questioned. The results presented here with berberine prompted us to examine the generality of the notion that inhibitors of mitochondrial function, including metformin, hinder mitogenic signaling through AMPK-dependent and independent mechanisms in a dose-dependent manner. We found that metformin inhibited mitogenic signaling (mTORC1, ERK and DNA synthesis) through an AMPK-dependent pathway when used at 1 mM and in PDAC cells cultured in physiological concentration of ambient glucose (5 mM). In this context, it will be of interest to examine whether berberine and metformin display synergistic effects with other molecules that act directly on AMPK, including A-769662 [91], [92]. However, at higher concentrations, metformin inhibited mitogenic signaling and DNA synthesis through an AMPK-independent mechanism. In support of this conclusion, AMPK knockdown prevented the increase in the phosphorylation of ACC at Ser^79^ and Raptor at Ser^792^ induced by metformin at either low or high doses. Remarkably, these results were obtained with metformin used at doses that induced either modest or pronounced declines in intracellular ATP levels, which were virtually identical to the decreases in ATP levels obtained in response to berberine (Fig. 3 B). It is plausible that the decline in ATP levels produced by higher doses of either berberine or metformin interferes with ATP-consuming processes required for anabolic metabolism and cell proliferation in an AMPK-independent manner. We therefore propose that berberine and metformin inhibit mitogenic signaling in PDAC cells through an AMPK-dependent pathway at low concentrations but act via AMPK-independent pathways in the same cells when added at higher doses. This conclusion provides a plausible explanation for apparently contradictory reports on the role of AMPK in the mechanism of action of berberine and metformin in other model systems and emphasizes the need of using detailed dose-response studies to define the anticancer mechanisms of action of agents that produce metabolic stress via inhibition of mitochondrial function.

In conclusion, our results raise the attractive possibility that treatment with berberine, a widely used agent used in traditional medicine, directly inhibits pancreatic cancer cell proliferation. Our mechanistic studies with berberine and metformin provide evidence in favor of a dose-dependent tumor suppressive role of AMPK in PDAC cells and offer the bases for novel therapeutic strategies for the treatment of pancreatic cancer, a devastating disease with limited survival option.

## Supporting Information

S1 Figure **Berberine inhibits mTORC1 signaling and ERK activation in PDAC cells stimulated with fetal bovine serum (FBS).** Cultures of PANC-1 cells were incubated in the absence or in the presence of increasing concentrations of berberine. Then, the cells were stimulated for 1 h with 2.5% FBS and lysed with 2X SDS-PAGE sample buffer. The samples were analyzed by SDS-PAGE and immunoblotting with antibodies that detect the phosphorylated state of S6K at Thr^389^, S6 at Ser^240/244^, and ERK at Thr^202^ and Tyr^204^. Immunoblotting with total S6K, S6 and ERK was used to verify equal gel loading.
(PDF)

S2 Figure **A769662 inhibits mTORC1 signaling and DNA synthesis in PANC-1 cells. A)** Cells were incubated without or with 50 mM A769662 or 1 mM metformin and stimulated with 5 nM neurotensin (NT) and 10 ng/ml insulin (Ins). Lysates were analyzed by SDS-PAGE and immunoblotting with antibodies that detect the phosphorylated state of ACC at Ser^79^, Raptor at Ser^792^, S6K at Thr^389^ and S6 at Ser^240/244^. Irrelevant lanes in the original autoradiograph were removed and relevant ones yuxtaposed (indicated by the vertical line). **B)** A769662 (50 mM) does affect mitochondrial membrane potential (fluorescence ratio) measured with JC-1 or reduces ATP levels. **C** Dose-dependent inhibition of [**^3^**H]-thymidine incorporation into DNA by increasing concentrations of A769662 in PANC-1 cells stimulated with neurotensin and insulin. **Image Editing:** Irrelevant lanes were removed (indicated by a thin, vertical black line) from the acquired digital images and flanking lanes juxtaposed using Adobe Photoshop.
(PDF)
