# Supplementary figures and images for: Dose-Dependent AMPK-Dependent and Independent Mechanisms of Berberine and Metformin Inhibition of mTORC1, ERK, DNA Synthesis and Proliferation in Pancreatic Cancer Cells
Source: PLoS One. 2014 Dec 10;9(12):e114573. doi: 10.1371/journal.pone.0114573 (PMC4262417; doi:10.1371/journal.pone.0114573)

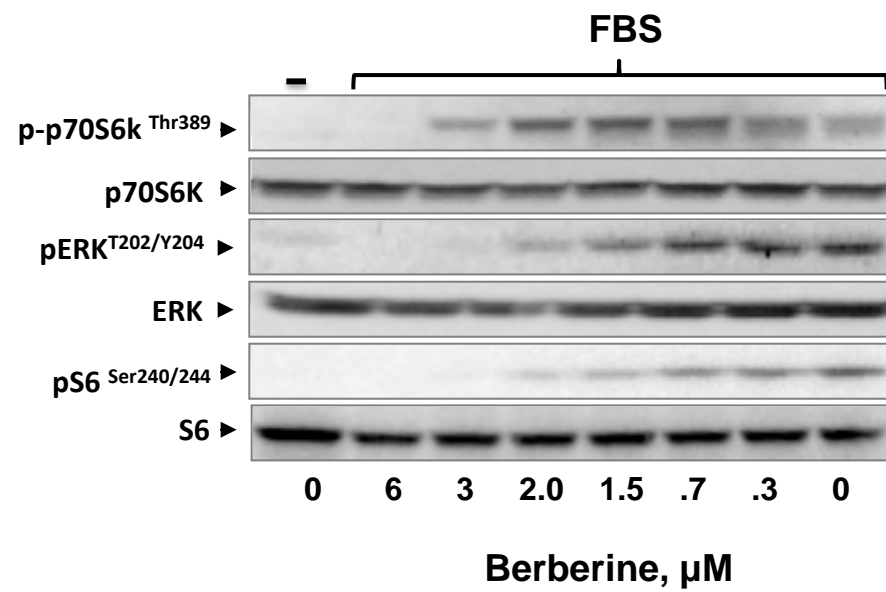

Supplementary Figure S1.

Supplement: S1 Figure — Berberine inhibits mTORC1 signaling and ERK activation in PDAC cells stimulated with fetal bovine serum (FBS). Cultures of PANC-1 cells were incubated in the absence or in the presence of increasing concentrations of berberine. Then, the cells were stimulated for 1 h with 2.5% FBS and lysed with 2X SDS-PAGE sample buffer. The samples were analyzed by SDS-PAGE and immunoblotting with antibodies that detect the phosphorylated state of S6K at Thr389, S6 at Ser240/244, and ERK at Thr202 and Tyr204. Immunoblotting with total S6K, S6 and ERK was used to verify equal gel loading. (PDF) [file pone.0114573.s001.pdf]

**A**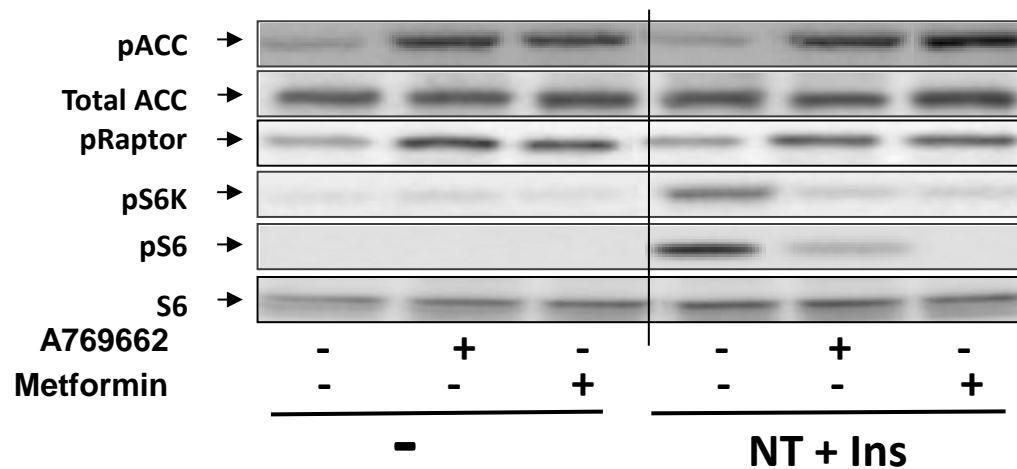**B**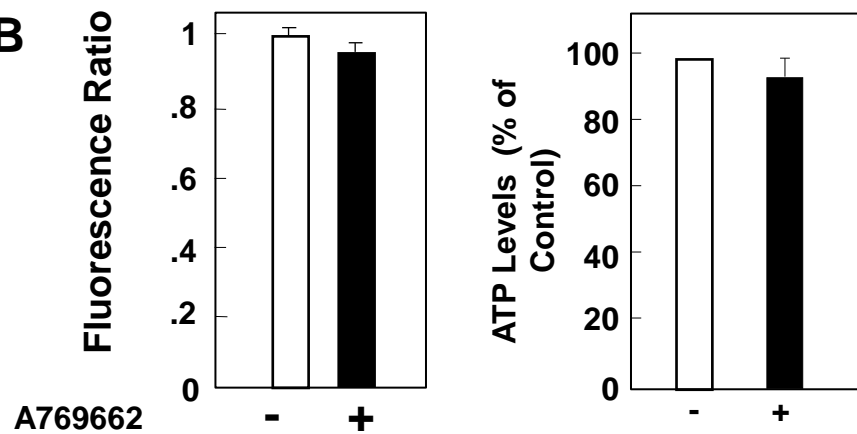**C**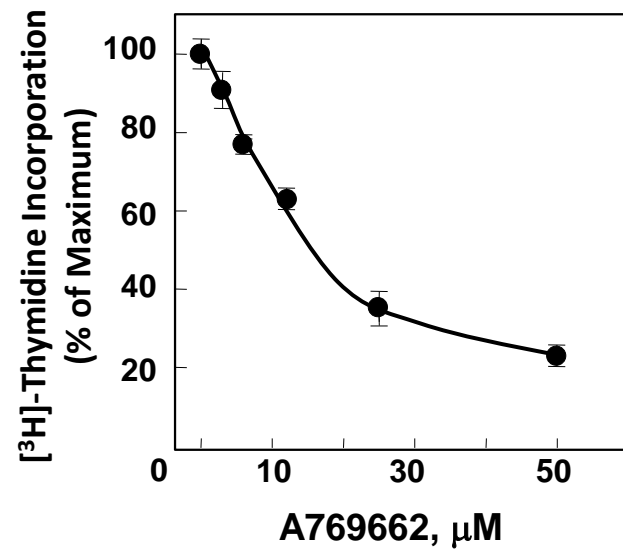

Supplementary Figure S2.

Supplement: S2 Figure — A769662 inhibits mTORC1 signaling and DNA synthesis in PANC-1 cells. A) Cells were incubated without or with 50 mM A769662 or 1 mM metformin and stimulated with 5 nM neurotensin (NT) and 10 ng/ml insulin (Ins). Lysates were analyzed by SDS-PAGE and immunoblotting with antibodies that detect the phosphorylated state of ACC at Ser79, Raptor at Ser792, S6K at Thr389 and S6 at Ser240/244. Irrelevant lanes in the original autoradiograph were removed and relevant ones yuxtaposed (indicated by the vertical line). B) A769662 (50 mM) does affect mitochondrial membrane potential (fluorescence ratio) measured with JC-1 or reduces ATP levels. C Dose-dependent inhibition of [3H]-thymidine incorporation into DNA by increasing concentrations of A769662 in PANC-1 cells stimulated with neurotensin and insulin. Image Editing: Irrelevant lanes were removed (indicated by a thin, vertical black line) from the acquired digital images and flanking lanes juxtaposed using Adobe Photoshop. (PDF) [file pone.0114573.s002.pdf]
